# Management of Transanal Recto-Anal Foreign Body in a School-Aged Child: A Report of a Rare Case

**DOI:** 10.7759/cureus.73120

**Published:** 2024-11-06

**Authors:** Takuma Iwai, Hiroshi Yoshida

**Affiliations:** 1 Gastroenterological Surgery, Nippon Medical School, Tokyo, JPN

**Keywords:** dangerous sexual practices, endoscopy, foreign body removal, rectal-anal foreign body, school-aged child, transrectal recto-anal foreign body

## Abstract

Transrectal recto-anal foreign bodies are occasionally encountered in routine practice, with sexual experimentation as the most common motivation for insertion. Although most patients with recto-anal foreign bodies are middle-aged, reports in younger patients are rare. In the present study, we encountered a case of recto-anal foreign body in a school-aged child. This report highlights some unique precautions for managing such cases in this population. The patient, a 12-year-old male, was referred to our department after a lip balm was inserted transanally, causing persistent discomfort. The patient exhibited no spontaneous abdominal pain or tenderness. The motive for inserting the foreign body was sexual interest. An abdominopelvic CT scan showed a foreign body that had entered the upper rectum. Lumbar anesthesia was administered, and the patient was positioned in the lithotomy position. Under a lower endoscope, one end of the foreign body was grasped with forceps and withdrawn into the lower rectum. The foreign body was removed with the assistance of an assistant performing anal dilatation. The patient was discharged the next day after confirming the absence of complications. Managing recto-anal foreign bodies in younger patients requires specific considerations, such as gentle handling to avoid damage due to stronger anorectal tonus. Moreover, it is crucial to assess potential social or psychological factors, including the possibility of abuse or bullying, that may underlie the insertion of the foreign body.

## Introduction

Transanal recto-anal foreign bodies are occasionally encountered in routine practice. Sexual experimentation or curiosity is the most common motivation for recto-anal foreign body insertion, and it is said to be on the increase in recent years [1,2]. Most patients are male, with most cases reported in middle-aged individuals [3,4]. A significant challenge in managing this condition is the delay in presentation, as many patients feel embarrassed and are reluctant to seek medical care. Despite this, recto-anal foreign bodies require careful management due to the risk of severe complications, such as gastrointestinal perforation and peritonitis, which may necessitate emergency surgery [5].

On the other hand, reports of recto-anal foreign bodies in young patients are rare, and there has been insufficient consideration of the precautions necessary for managing such cases.

## Case presentation

A 12-year-old boy with no prior medical history was presented. Two days prior, the patient inserted a foreign body (lip balm) transanally, and it became lodged in his rectum. The patient presented to the department because he had difficulty defecating and continued to experience discomfort. The motive for inserting the foreign body was sexual interest.

**Figure 1:**
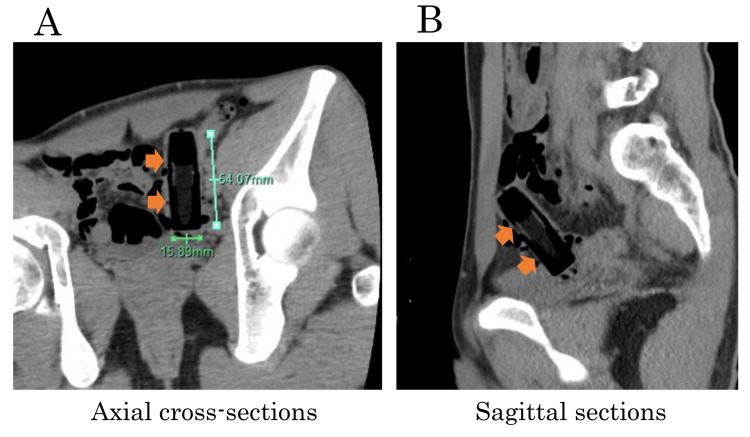
CT findings Abdominopelvic CT shows no evidence of gastrointestinal disturbance, as there is no free air, ascites, or inflammation around the intestinal tract. Axial (A) and sagittal (B) sections of the CT are shown. A rod-shaped foreign body (orange arrow) has strayed into the upper rectum.

On admission, the patient was in stable general condition with a blood pressure of 118/78, a pulse rate of 60/min, and a body temperature of 36.4 °C. His abdomen was flat and soft, without spontaneous pain or tenderness. There were no external injuries or bruises. Blood samples showed no abnormalities. An abdominopelvic CT scan showed no free air, ascites, or inflammatory changes around the intestinal tract and no signs of gastrointestinal damage. The CT scan revealed a rod-shaped foreign body that had entered the upper rectum (Figure 1, orange arrow).

On rectal examination, the lower end of the moving foreign body in the rectum is palpable but difficult to grasp. The strong anorectal tonus made rectal manipulation painful, leading to the decision to remove the foreign body under anesthesia.

**Figure 2:**
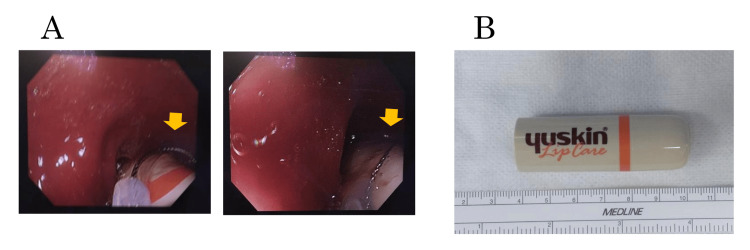
Extraction of foreign bodies The foreign body was grasped with snare forceps under a lower endoscope and pulled close to the anus (A, yellow arrow). The extracted foreign body is shown in panel B.

Lumbar anesthesia plus intravenous sedation was administered, and the patient was positioned in the lithotripsy position. The foreign body was grasped with snare forceps under a lower endoscope, withdrawn into the lower rectum, and removed with the assistance of an assistant assisting with anal dilatation (Figure 2).

There was no obvious rectal mucosal damage or other complications after the procedure.

## Discussion

Recto-anal foreign bodies are more commonly seen in middle-aged men [5,6]; however, school-aged cases present distinct challenges. The physical aspect is that the insertion of the foreign body is not a regular occurrence and the anal tonus is strong; the psychological aspect is that it is embarrassing and difficult to discuss with others; and the social aspect is the possibility of abuse [7]. It is important to recognize that children require distinct treatment approaches compared to adults.

Regarding physical aspects

If the anal sphincter function is impaired at a young age, it will have a significant impact on the long-term quality of life [8], so protective treatment is required. The medical staff should avoid excessive dilatation of the anus and try to perform non-invasive procedures such as anesthesia to relieve tension and endoscopic procedures. If there is any evidence of damage to the rectum or anus, it is important not to forget to carry out a functional assessment and follow-up.

Regarding mental and social aspects

In younger cases, the possibility of bullying or sexual abuse should be considered, and physical checks and careful interviewing for evidence of abuse are important [7,9]. This precaution also applies to older cases [10].

Interviews should be conducted before and after foreign body removal, as the responses may differ depending on the patient’s comfort and state of mind post-procedure. Determining the most suitable staff member to conduct these interviews is also critical. For example, should the doctor or nurse integrate questioning naturally into the examination process, or should a social worker facilitate a more structured interview post-procedure? It may also be preferable to conduct interviews in a private setting without the presence of family members, allowing the patient to feel secure when sharing sensitive information. Additionally, interviewing the family separately can help verify if their accounts align with the patient's, adding another layer of scrutiny.

If abuse is suspected, it may be necessary to involve child protection services or law enforcement. Similarly, if bullying is suspected, sharing information with the school or school board could help address the issue from an educational perspective.

In addition, once the foreign body has been removed, medical staff should provide the patient with a clear, age-appropriate explanation of the physical risks associated with inserting objects into the anus. In our case, the doctor and nurse conducted a thorough interview. No signs of abuse or bullying were found, and the patient was discharged after receiving an educational explanation.

Finally, the recent spread of smartphones has increased the risk that children might encounter inappropriate sexual content online. Such environmental factors may influence children's behavior, underscoring the importance of health professionals remaining aware of these broader contextual elements when treating young patients.

## Conclusions

Young patients with recto-anal foreign bodies are rare. Key considerations in the treatment of young patients include: (i) they require more gentle treatment than middle-aged patients, in whom foreign body insertion is a regular occurrence and the anorectal tonus is loose; and (ii) careful physical examination and interview are necessary, considering the possibility of bullying or abuse as a cause of foreign body insertion.
